# Bibliographical Notices

**Published:** 1844-03-23

**Authors:** 


### BIBLIOGRAPHICAL NOTICES.

*Lectures on the more important Diseases of the Thoracic and Abdominal Viscera, Delivered in the University of Pennsylvania.* By N. Chapman, M. D., Professor of the Theory and Practice of Medicine, etc. etc. Philadelphia: Lea and Blanchard. 1844. Octavo. pp. 383.

"Essentially does this work consist of lectures, which have been publicly delivered," is the initiatory remark of the author in his preface to this publication. It is known, probably, to most of our readers, that the distinguished author is not among the *changeable* people of our day, and at his period of life, especially, most men are little liable to be carried away by the novelties and innovations of the younger and more restless spirits of the age. No one will be surprised, therefore, who has sat, like ourselves, at the feet of this Gamaliel, to find the language and the views he was wont to listen to in the days of his youth, put forth in this latter work with all the earnestness of long cherished conviction. Beside the opportunities afforded of becoming acquainted with the author's opinions, in the manner alluded to, some of the articles contained in the work "have previously appeared in one or more of our Medical Journals," and must, therefore, be familiar to a large portion of our readers. On this account, as well as our restricted limits, our notice of it will be confined to a few passing remarks.

The subjects treated of in this work, although not numerous, are among the more interesting of those embraced in the practice of medicine. On some points the author's opinions, if not very recently formed, are at least *peculiar,* and the style in which they are conveyed eminently so. One of his peculiar opinions is expressed in the following paragraph: —"As prone to consumption as most parts of the world, scrofula is seldom or never generated among us; the disease which we occasionally see being chiefly in foreigners or their immediate descendants."—*Page* 25.

When we consider that a large portion of the inhabitants of this country are "foreigners or their immediate descendants," it is not strange that a very large number of the cases of disease of every type should happen among such; but that scrofula does not occur among the natives and descendants of natives, is not at all accordant with our experience, or the experience of any respectable member of the profession with whom we have conversed on the subject.

In regard to percussion and auscultation, as means of discovering the condition of the organs of the chest, we have read with surprise the following remark:—"Both it (percussion) and auscultation seem of late to be gradually losing much of their former cordiality of support, and by many are treated contemptuously. Details I cannot give."—*Page* 40.

We had thought that these means of diagnosticating diseases of the thorax in particular, were really growing in favour with the profession as they become better understood, and were quite of opinion that they were treated contemptuously only by those who found it easier to sneer than to learn. For ourselves we make no great pretension to skill in their use, but we have seen enough to cause us to repose confidence in the conclusions of some whose opportunities have been greater than our own for acquiring tact in their employment, and we are glad to discover, from other passages, that our author still regards them "as important auxiliaries in the exploration of diseases of the chest."

Beside these and some other peculiarities of a like character, we find also occasional errors, some of which are rather unaccountable. For instance, in speaking of the excellent and accomplished Dr. Baillie, he is called *Sir* Mathew Baillie. This we think is a mistake; the title we believe was never conferred on him, although it often has been on men much his inferiors, in and out of the profession.

Page 153, referring to John Hunter, the author remarks:—"Naturally very irascible, he got into a quarrel with Gunning, an old wayward surgeon, one of his colleagues at St. Georges Hospital, *about the treatment of a compound fracture,* for whom entertaining great contempt, his anger became exasperated to rage, and down he fell, and soon expired. This anecdote I derived from the late Professor Physick, then one of his pupils, who, I think, witnessed the occurrence." This statement contains several errors. In the first place, Dr. Physick, according to the memoir of his life and character by his son-in-law, Dr. Randolph, "returned to his native country in Sept., 1792," and Hunter died in Oct., 1793,—13 months after Dr. P.'s departure from Europe. In regard to Hunter, the manner of his death was as follows:—The Governors of St. Georges Hospital, of which he was one of the surgeons, had adopted a regulation by which none were admitted as pupils without previous medical instruction. Two young men from the country applied to Hunter to be admitted as pupils to the Hospital, under him, without such preparation. Hunter endeavoured to procure their admission, contrary to the regulation referred to, and for that purpose presented the memorial of the young men to the board, and, "in the course of his remarks on the occasion, he made some observations which one of his colleagues thought it necessary instantly and flatly to contradict. Hunter immediately ceased speaking, retired from the table, and, struggling to suppress the tumult of his passion, hurried into the adjoining room, which he had scarcely reached, when, with a deep groan, he fell lifeless into the arms of Dr. Robertson, one of the Physicians of the Hospital, who chanced to be present."—*Life of John Hunter, by Drewry Ottley, Phidadelphia Ed.*

Among his other dislikes in regard to modern improvements, real and imaginary, the author exhibits a proper abhorrence of Thompsonism and Homœopathy. In reference to the latter, after some remarks on the absurdity of it, both in theory and practice, he thus discourses of the knavery of its pretenders:—"Nor longer ought it to be concealed that those mercenary miscreants, perceiving a loss of public confidence in the utter inertness of the original practice, and particularly in the avowed infinitesimal doses of medicines, are whirling around into the opposite extreme—now resorting to the most active, and in exorbitant quantities. The articles to which they are at present devoted—arsenic, veratria, and aconite—are the most deleterious, when incautiously directed, of the whole Materia Medica. But 'fools rush in where angels fear to tread.'" To all this we say amen! and right glad are we to have such testimony on the subject.

The remarks of the author on the incurability of confirmed consumption, and the baseness of those who proclaim the contrary for filthy lucre, are worthy of all praise; nor are his observations less deserving of attention in reference to the nature and influence of the climate in the United States, on pulmonary diseases:—"Many still entertain the opinion that it is not prudent to confine a patient anywhere within the boundaries of the United States during winter. Do such persons remember that our limits are nearly as wide as those of all Europe, and embrace as great diversity of climate? Taken as a whole, I believe it to be the best in the world, and portions of it, I cannot doubt, are as well adapted to the end now in view, as is attainable elsewhere. The contrary estimate of it was originally derived from the prejudices of our early settlers, since strengthened by foreigners who delight to do us injustice, and confirmed by our own acquiescence. That of Europe, which we are accustomed to commend, is the creation of a poet's fancy. 'Distance lends enchantment to the view.' There is no part of it in which the winter season is not, in reality, most horrible, and that of Britain and France, scarcely endurable."

It is indeed quite too much the custom with our physicians to direct their patients to other countries and climates when ordinary means fail. Too often "the change is for the worse, not the better;" and, instead of obtaining relief, the poor sufferer is made to endure the the fatigue of travelling, the privation of all the comforts of home, and the thousand other annoyances inseparable from the change. And all for what? That his physician may be relieved from the irksome duty of attendance upon an incurable case, and the hope, against all experience, that each case may be more fortunate than all which have preceded it.

Notwithstanding the views of the author contained in the present work must be familiar, as we have said, to a large portion of the profession in this country, his high reputation, and the distinguished position he has long held as a teacher in the oldest school of medicine on the Continent, will secure for the present volume an extensive sale and very general perusal.

---

*An Elementary Treatise on Human Physiology, on the basis of the Precis Elementaire De Physiolgie.* Par F. Magendie, &c. *Fifth edition*, 1838. *Translated, Enlarged, and illustrated with diagrams and cuts. Especially designed for the use of students of medicine.* By John Revere, M. D., Professor of the Theory and Practice of Medicine in the University of the city of New York.

The name of Magendie is familiar to every tyro in physiology—since Haller, none has been more so. As an experimenter, he has displayed great perseverance, boldness, and originality. While, however, he has in this way added some important facts to the science, much that he has advanced as ascertained results, has not been confirmed by other and equally competent observers. A less overweening confidence in himself, with a juster appreciation of the labours of other people, might, in some instances at least, have saved him from the errors into which he has fallen. The work before us is emphatically Magendie's Physiology, and those who read it will discover that it is an exposition of *his* particular views, not an impartial account of the science as at present received and acknowledged by the great majority of the profession. By the admirers of the author, it is praised as an *original* work; and perhaps it is as well entitled to be so regarded as any recent treatise on a science which has been long cultivated. To our mind, however, this is poor praise. The man who writes an original work on such a subject—that is, one composed wholly of materials supplied by himself,—must write a very small book, or a very large amount of nonsense. But Magendie's work, with all its faults, and it has many, does not deserve to be thus condemned. It is not *all* original; and that which is not, is far from being always the least valuable.

The additions by the Editor and Translator, supply some great deficiencies in the work, and add much to its value; but we think he has failed in his "aim to present a system of human physiology, which shall exhibit in a clear and intelligible manner the actual state of the science, and adapted to students of medicine in the United States"—we know in fact of no recent work on the subject that does not better represent the *actual state* of the science, or that is not better adapted to students of medicine, either in the United States or any where else. This, however, is the Editor's mistake, not his fault. A very little more labour than he has bestowed on his author, would have enabled him to produce a far more systematic work, exhibiting the "actual state of the science," and every way better "adapted to students of medicine."

---

*Drawings of the Anatomy of the Groin: with Anatomical remarks.* By W. Darrach, M.D., Professor of the Principles and Practice of Medicine, in the Medical Department of Pennsylvania College; Fellow of the College of Physicians of Philadelphia, &c. &c. Philadelphia: Lindsay & Blakiston, 1844. Second Edition.

"The original drawings of this publication were executed by M. Chasal of Paris, from dissections made by the Author, in the *Pavilions de l'Ecole de Medecin*, during the winter of eighteen hundred and twenty. Those of the present edition of this work,—reduced in size,—have been executed by Mr. M. S. Weaver of Philadelphia."

The work consists of four plates, very well executed, representing the anatomy of the groin, and calculated to give to students and others right conceptions of the parts concerned in hernia.

Beside the plates, the work contains one hundred and twenty-seven pages of explanatory text, with appropriate references.

